# A new species of *Chlamydia* isolated from Siamese crocodiles (*Crocodylus siamensis*)

**DOI:** 10.1371/journal.pone.0252081

**Published:** 2021-05-27

**Authors:** Somjit Chaiwattanarungruengpaisan, Metawee Thongdee, Songtham Anuntakarun, Sunchai Payungporn, Nlin Arya, Apichart Punchukrang, Pongrama Ramasoota, Sombat Singhakaew, Thassanant Atithep, Ladawan Sariya

**Affiliations:** 1 The Monitoring and Surveillance Center for Zoonotic Diseases in Wildlife and Exotic Animals, Faculty of Veterinary Science, Mahidol University, Nakhon Pathom, Thailand; 2 Center of Excellence in Systems Biology, Research Affairs, Faculty of Medicine, Chulalongkorn University, Bangkok, Thailand; 3 Department of Biochemistry, Faculty of Medicine, Chulalongkorn University, Bangkok, Thailand; 4 Department of Preclinic and Applied Animal Science, Faculty of Veterinary Science, Mahidol University, Nakhon Pathom, Thailand; 5 Faculty of Agricultural Technology, Songkhla Rajabhat University, Songkhla, Thailand; 6 Center of Excellence for Antibody Research and Department of Social and Environmental Medicine, Faculty of Tropical Medicine, Mahidol University, Bangkok, Thailand; 7 Department of Biology, Faculty of Science, Mahidol University, Bangkok, Thailand; 8 Frontier Research Center, Vidyasirimedhi Institute of Science and Technology, Rayong, Thailand; University of Zurich, SWITZERLAND

## Abstract

*Chlamydia* is a known pathogen in both saltwater and freshwater crocodiles. However, the exact species/strain has not been clearly identified. In this study, we successfully cultivated Siamese crocodile *Chlamydia* in McCoy cells at a temperature of 30°C. Electron microscopy; phylogeny based on nine conserved taxonomically informative markers, on *ompA*, or on seven housekeeping genes; and whole-genome sequencing and analysis of the isolate confirmed the identity of the isolate as a new member of the genus *Chlamydia*, a new species that we name *Chlamydia crocodili*.

## Introduction

The family *Chlamydiaceae* is composed of a single genus, *Chlamydia*, containing 15 known species (*C*. *abortus*, *C*. *avium*, *C*. *buteonis*, *C*. *caviae*, *C*. *felis*, *C*. *gallinacean*, *C*. *ibidis*, *C*. *muridarum*, *C*. *percorum*, *C*. *pneumonia*, *C*. *poikilothermis*, *C*. *psittaci*, *C*. *serpentis*, *C*. *suis*, and *C*. *trachomatis*) and 2 *Candidatus* species (*Ca*. *C*. *corallus* and *Ca*. *C*. *sanzinia*) [1–4]. *Chlamydiaceae* has been identified in various hosts, such as birds, reptiles, and mammals including humans [5]. In crocodiles (*Crocodylus niloticus* and *C*. *porosus*), *Chlamydia* was previously reported in South Africa and Papua New Guinea [6, 7]. However, the species/strain of this *Chlamydia* has not been clarified. In Thailand, the first outbreak of *Chlamydia* in Siamese crocodiles (*C*. *siamensis*) was reported in 2012 [8]. Molecular evidence based on 16S ribosomal RNA (rRNA), the 16S/23S rRNA gene, and the major outer membrane protein (*ompA*) gene demonstrated that this outbreak was due to a novel species of *Chlamydia* [8]. Regarding the clinical signs of infected crocodiles, they could be asymptomatic, or show kyphoscoliosis in juveniles, conjunctivitis, pharyngitis, ascites, depression, anorexia, and death [7–9]. In some *Chlamydia* outbreaks, co-infection with *Aeromonas sobria* [9] and herpesvirus was identified [10]. In this study, we successfully isolated *Chlamydia* from infected Siamese crocodiles using McCoy cells and characterized its whole-genome sequence. The overall results suggested that the strain is related to a new species of *Chlamydia*, which we named *C*. *crocodili*.

## Materials and methods

### Ethics approval

This study was approved by the Animal Care and Use Committee (Protocol No. MUVS-2016-08-30 and MUVS-2018-08-37) and the Institutional Biosafety Committee (Protocol No. IBC/MUVS-B-004/2561) of the Faculty of Veterinary Science, Mahidol University, Nakhon Pathom, Thailand.

### Isolation and growth characterization of Siamese crocodile *Chlamydia*

The isolation of *Chlamydia* was attempted from a PCR-positive liver tissue sample of an infected Siamese crocodile. This infected Siamese crocodile’s carcass was obtained from Trang Province, Thailand, in 2018. The liver tissue sample was collected and stored at −80°C until use. The *Chlamydia* isolation was performed following a modified version of a previously reported protocol [11]. A full step-by-step protocol for *Chlamydia* isolation has been deposited in the protocols.io repository (https://dx.doi.org/10.17504/protocols.io.btcqnivw). The liver tissue sample was homogenized in sucrose/phosphate/glutamate buffer containing 500 μg/ml streptomycin, 500 μg/ml vancomycin, 50 μg/ml gentamycin, and 2.5 μg/ml fungizone and left at 4°C for 72 h. Prior to inoculation into McCoy cells (ATCC^®^ CRL-1696^™^; American Type Culture Collection, VA, USA), the homogenized tissue sample was centrifuged at 250×g for 10 min and the obtained supernatant was collected. Monolayers of McCoy cells were prepared in 12-well plates by cultivating overnight in M199 (Gibco BRL Life Technologies Inc., NY, USA). The cells were infected using an inoculum to reach infectivity of approximately 80% at 4–5 days post-infection. The incubation medium was composed of M199 (Gibco BRL Life Technologies Inc., NY, USA) containing 0.5 μg/ml cycloheximide and supplemented with 10% heat-inactivated fetal calf serum (Gibco BRL Life Technologies Inc., NY, USA), 0.2% NaHCO_3_, 10% glucose, 1× L-glutamine, 100 μg/ml streptomycin, 100 μg/ml vancomycin, 10 μg/ml gentamycin, and 1 μg/ml fungizone. Infected monolayers were centrifuged for 1 h at 1,000×g and 25°C. After centrifugation, duplicate infected cultures were incubated at 30°C and 37°C in an atmosphere of 5% CO_2_ for an additional 1 h before replacing the inocula with fresh incubation medium. Duplicate infected cultures were further incubated at 30°C and 37°C in an atmosphere of 5% CO_2_ and preliminary evaluations were performed at 24, 48, 72, 96, and 120 h post-infection (hpi). At the indicated time points, infected cells were further processed for quantitative real-time PCR, cytopathic effect (CPE) observation by phase contrast microscopy, and indirect immunofluorescence microscopy assay (IFA).

### Preparation of standard plasmid and quantitative real-time PCR

To prepare the standard plasmid, the 16S rRNA fragment was amplified by *Chlamydiaceae* family-specific 16S rRNA gene primers [12] and purified using QIAquick gel extraction kit (QIAGEN, Hilden, Germany). The 16S rRNA fragment was ligated into pGEM^®^-T easy vector (Promega Corporation, WI, USA) which was then transformed into *E*. *coli* DH5α competent cells. The plasmid was purified using the QIAprep spin miniprep kit (QIAGEN, Hilden, Germany) and the concentration was measured at 260 nm using NanoDrop One (Thermo Fisher Scientific, MA, USA) to calculate the DNA copy number.

For quantitative real-time PCR, 200 μl of cell culture supernatant at each indicated time point was subjected to DNA extraction using the Genomic DNA Mini kit (blood and cultured cells) (Geneaid, Taipei, Taiwan), in accordance with the supplier’s recommendations. Extracted DNA was examined using real-time PCR based on *Chlamydiaceae* family-specific 16S rRNA gene primers [12]. The quantitative real-time SYBR Green I-based PCR amplification mixture contained 12.5 μl of 2× QuantiTect SYBR Green PCR master mix (QIAGEN, Hilden, Germany), 0.5 μM of each primer, and 1 μl of DNA template. The reaction was performed in a total volume of 20 μl and was carried out in the Chromo4^™^ real-time PCR instrument (Bio-Rad^™^, CA, USA) under the following conditions: 15 min at 95°C to activate DNA polymerase, followed by 40 cycles of 15 s at 95°C and 45 s at 56°C. Tenfold serial dilutions of standard plasmid and no template control were also performed in each run. All samples were tested in triplicate. After PCR cycling, melting curve analysis was performed on the PCR-amplified product to measure the specificity of PCR at 60°C–95°C with continuous fluorescence measurement with each 1°C increase. A mean cycle threshold (Ct value) of <38 was considered positive, and was used to calculate the corresponding copy number per microliter compared with a standard curve.

### Indirect immunofluorescence assay (IFA)

IFA was performed using a modified version of a previously reported method [13]. McCoy cells grown on glass coverslips (∅ 15 mm) in 12-well plates were inoculated with crocodile *Chlamydia* from the third cell culture passage. Infected cells were centrifuged and incubated as described above. At 4–5 days post-infection, infected cells were fixed with 4% paraformaldehyde for 24 h. Fixed samples were rinsed with phosphate-buffered saline (PBS) and permeabilized with 0.2% Triton X-100 in PBS for 30 min at 37°C. Samples were washed 3 times with PBS and then blocked with 1% BSA for 30 min at 37°C. To visualize chlamydial inclusions, coverslips were incubated with 1:100 diluted *Chlamydiaceae* family-specific mouse monoclonal antibody directed against the chlamydial lipopolysaccharide (cat. no. sc-58106, Santa Cruz Biotechnology, Inc., TX, USA) and 1:200 diluted Alexa Fluor 594-conjugated secondary goat anti-mouse antibody (cat. no. A11005, Life Technologies, CA, USA). DNA was labeled with 2 μg/ml 4′,6-diamidino-2′-phenylindole dihydrochloride (DAPI) (Molecular Probes, OR, USA). Coverslips were mounted onto microscope glass slides. Images were taken using a Leica DMi8 inverted fluorescence microscope (Leica Microsystems, Wetzlar, Germany) and analyzed with the Leica application suite X (LASX) imaging software (Leica Microsystems, Wetzlar, Germany).

### Transmission electron microscopy (TEM)

The morphology of the chlamydial strain isolated from Siamese crocodile was demonstrated using TEM [14]. McCoy cell monolayers prepared in a six-well culture plate were infected with *Chlamydia* from the third cell culture passage at 30°C, as described above. Infected cells were harvested at 96 hpi. Cells were fixed in 2.5% glutaraldehyde, scraped off the culture plate, and centrifuged at 250×g for 10 min. Cell pellets were washed with 0.5 M phosphate buffer (pH 7.2) and post-fixed with 1% osmium tetroxide. Specimens were then washed and dehydrated using 30%, 50%, 70%, 90%, and 100% acetone in series, for 10 min each. This was followed by infiltration with increasing concentrations of Araldite^®^ 502 epoxy resin (Sigma-Aldrich, Missouri, USA) and polymerized at 45°C and 60°C for 2 days each. Ultra-thin sections (80 nm) were cut using a Leica Ultracut R microtome (Leica Microsystems Inc., Wetzlar, Germany), mounted on copper grids, counterstained with 4% uranyl acetate and 1% lead citrate, and examined under a transmission electron microscope (JEM-ARM200F; JEOL Co. Ltd., Tokyo, Japan) operated at 200 kV. Digital images were collected using an OneView camera (Gatan Inc., CA, USA).

### Whole-genome sequencing, mapping, assembly, and annotation

Next-generation sequencing library preparations were constructed following the manufacturer’s protocol. Sequencing and analysis were carried out in a 2 × 150 paired-end fashion on an Illumina HiSeq instrument (Illumina, CA, USA). Prodigal (for prokaryotes) [15] gene finding software was used for finding coding genes in bacteria. Transfer RNAs (tRNAs) were detected in the genome using the program tRNAscan-SE [16] with the default parameters. rRNAs were identified using RNAmmer [17]. The coding genes were annotated with the National Center for Biotechnology Information (NCBI) nr database by BLAST. The proteins encoded by the genes were subjected to phylogenetic classification using the Clusters of Orthologous Groups database. The sequences of the *Chlamydia* and its plasmid obtained in this study were submitted to GenBank, with accession numbers CP060791 and CP060792, respectively.

### Phylogenetic tree and genomic analysis

The concatenated sequences of nine protein-coding phylogenetic markers (DNaA, SucA, Hyp325, FabI, RpoN, Ftsk, PepF, Adk, and HemL) [18], the *ompA* gene, and seven concatenated housekeeping genes (*gatA*, *oppA*, *hflX*, *gidA*, *enoA*, *hemN*, and *fumC*) [19] were aligned with the MAFFT program (version 7.450) [20] with the default parameters. The phylogenetic tree was generated by MEGA7 version 7.0.21 [21] with the Maximum Likelihood method and compared with the corresponding gene sequences from other members of the *Chlamydiaceae* family. The best substitution model with the lowest BIC scores (Bayesian Information Criterion) was used for each phylogeny construction. Pairwise amino acid sequence identities of the nine phylogenetic markers and nucleotide sequence identities of 16S and 23S rRNA were calculated based on the MAFFT alignment using the Sequence Identity and Similarity program (http://imed.med.ucm.es/Tools/sias.html). Digital DNA–DNA hybridization (dDDH) analysis and Genome BLAST Distance Phylogeny (GBDP) were performed at the Type (Strain) Genome Server (TYGS), a free bioinformatics platform available at https://tygs.dsmz.de, for whole-genome-based taxonomic analysis [22]. The average nucleotide identity (ANI) between two genomes was calculated using the ANI calculator available at http://enve-omics.ce.gatech.edu/ani/ [23].

## Results

### Isolation and growth characterization

**Fig 1:**
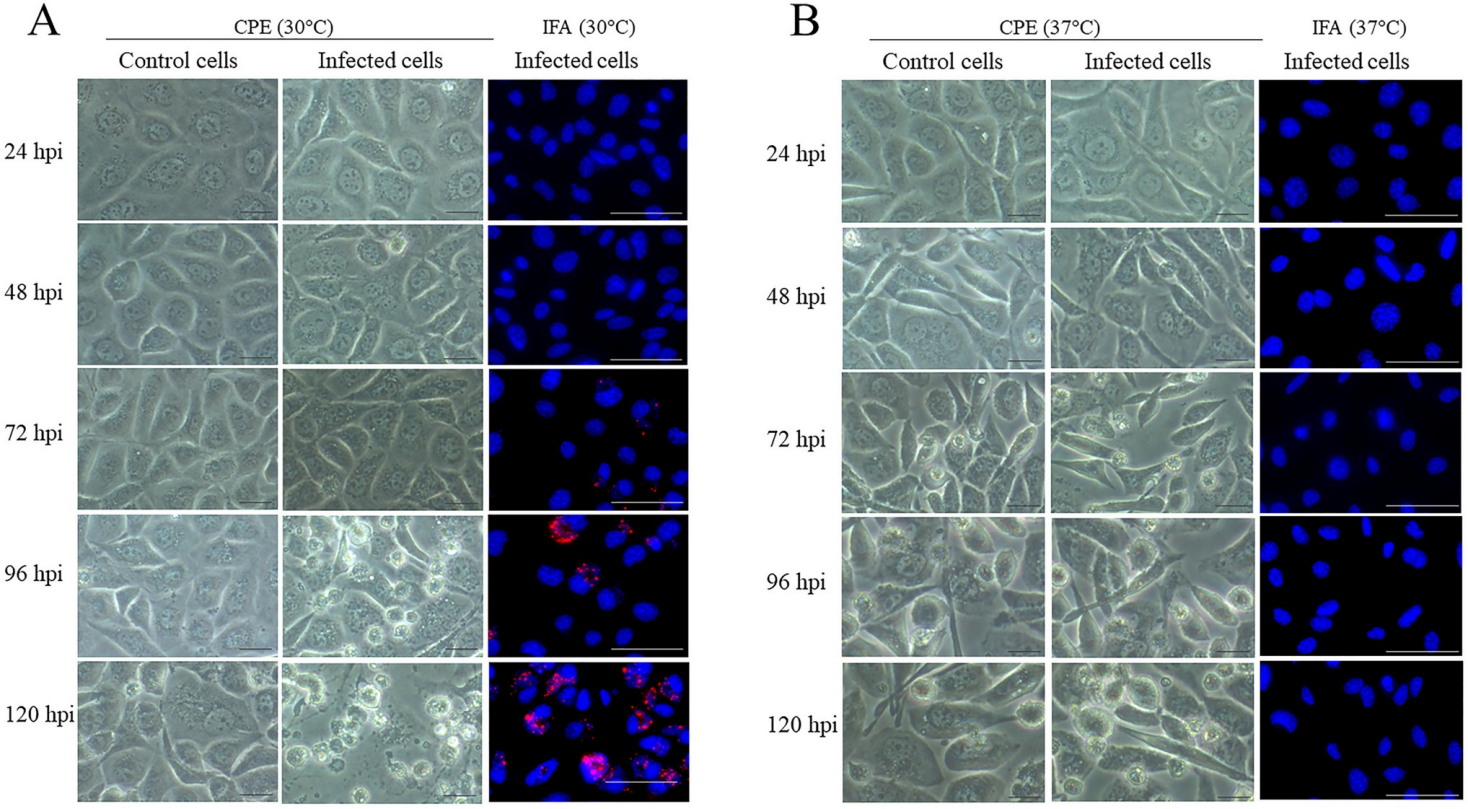
CPE and IFA of McCoy cells after Siamese crocodile *Chlamydia* infection. (A) McCoy cells were infected with *Chlamydia* at 30°C. (B) McCoy cells were infected with *Chlamydia* at 37°C. CPE of uninfected (control cells) and infected McCoy cells was observed by phase contrast microscopy at 32× magnification (scale bar = 10 μm). For the IFA test, infected McCoy cells were detected with *Chlamydiaceae* family-specific mouse monoclonal antibody and observed by fluorescence microscopy at 40× magnification (scale bar = 50 μm). CPE and IFA at each temperature were observed at 24, 48, 72, 96, and 120 hpi.

The infection efficiencies of the Siamese crocodile *Chlamydia* in McCoy cells, at incubation temperatures of 30°C and 37°C, were compared by quantitative PCR, CPE observation, and IFA after 0, 24, 48, 72, 96, and 120 hpi. Upon incubation at 30°C, DNA quantities from culture supernatant of infected cells continuously increased at 72, 96, and 120 hpi (Table 1). Structural changes in McCoy cells by the appearance of CPE could be observed at 72, 96, and 120 hpi when compared with the control cells (Fig 1A). These results indicated that the *Chlamydia* could infect and multiply in McCoy cells. The IFA test showed that the bacteria formed a cytoplasmic inclusion body in infected cells (Fig 1A). Upon cultivation at 37°C, the amount of DNA from infected cells did not increase with increasing infection time (48–120 hpi) (Table 1). CPE did not appear and the IFA result was negative at 37°C (Fig 1B). The results indicated that the temperature of 30°C was better for the growth and multiplication of Siamese crocodile *Chlamydia* than 37°C.

**Table 1:** Comparison of incubation temperature for *C*. *crocodili* isolation in McCoy cells at 0, 24, 48, 72, 96, and 120 hpi by measuring DNA quantity.

Incubation temperature/ hpi	Mean Ct	Mean quantity/μl DNA	Mean quantity/ml culture supernatant
Inoculum	23.75	1.74E+06	2.61E+08
30/0	UD.		
30/24	30.89	1.68E+04	2.52E+06
30/48	30.10	2.81E+04	4.22E+06
30/72	25.83	4.52E+05	6.78E+07
30/96	23.87	1.61E+06	2.41E+08
30/120	21.27	8.73E+06	1.31E+09
37/0	UD.		
37/24	UD.		
37/48	31.25	1.34E+04	2.00E+06
37/72	30.32	2.45E+04	3.67E+06
37/96	30.45	2.25E+04	3.37E+06
37/120	30.61	2.02E+04	3.03E+06

UD. = under the limit of detection.

### Ultrastructural analysis of *Chlamydia*-infected cells

**Fig 2:**
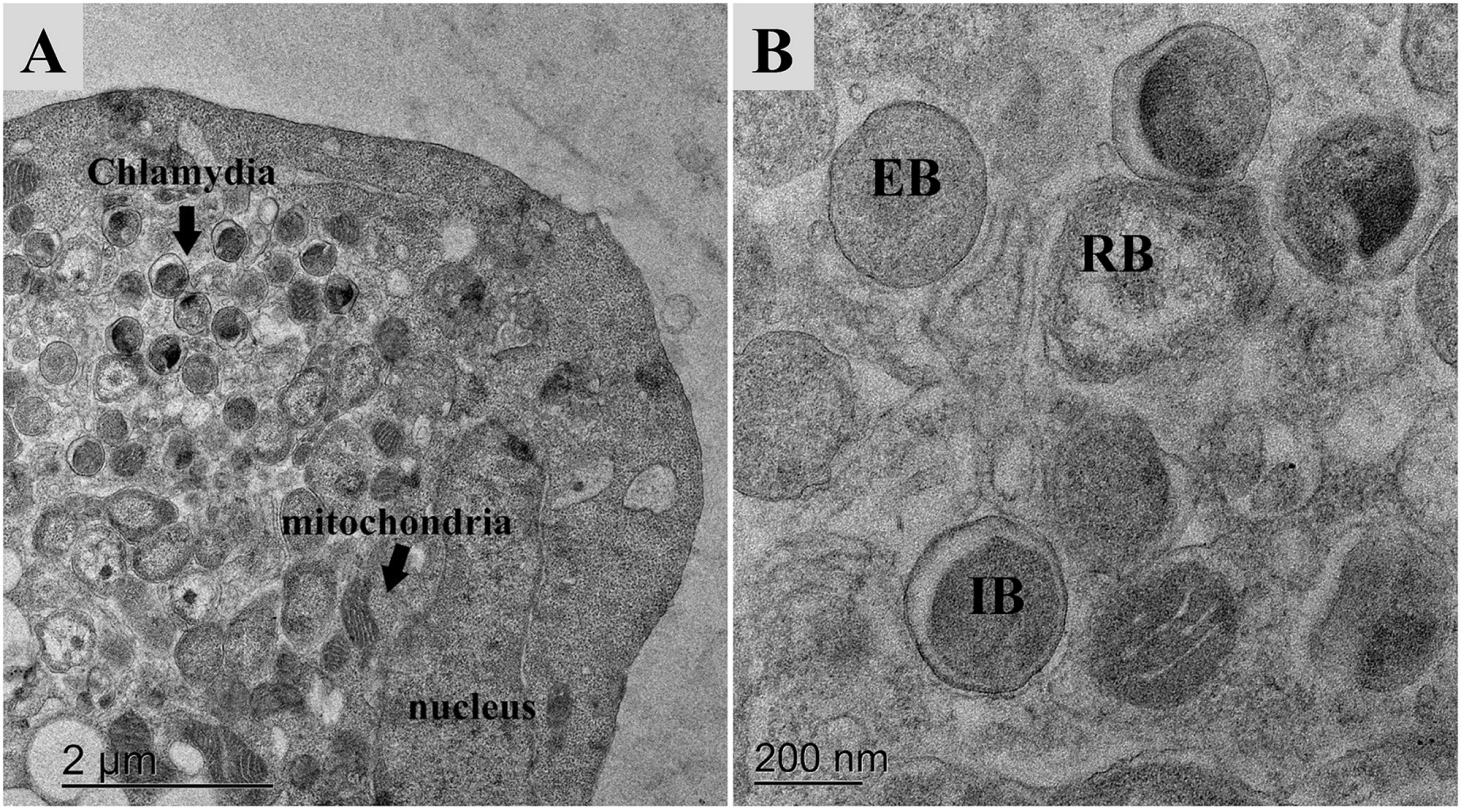
Electron microscopic images of McCoy cells infected with Siamese crocodile *Chlamydia*. (A) *Chlamydia* accumulated in the cytoplasm of the infected McCoy cells. (B) Typical forms of *Chlamydia* were observed: EBs, RBs, and IBs.

Ultrastructural analysis of McCoy cells infected with Siamese crocodile *Chlamydia* was performed at 96 hpi and 30°C. As shown in Fig 2, the electron-dense elementary bodies (EBs) and electron-lucent reticulate bodies (RBs) were observed in the cytoplasm. EBs were typically spherical with 0.2–0.39 μm diameter, a thick cell wall, dense nucleoid, and clear periplasm. RBs were larger than EBs, with 0.45–1.07 μm diameter, and were typically pleomorphic with a thin cell wall. RBs also appeared with a loosely distributed, somewhat granular cytoplasm lacking a condensed nucleoid. Moreover, intermediate bodies (IBs) showing a smaller condensed nucleoid than EBs were also observed.

### Genome characterization

Whole-genome sequencing and analysis showed that the total read count and count mapped for genome mapping were 17,910,306 and 8,694,474 (48.54% of mapped reads), respectively, with 100% genome coverage and 2,096.01 average sequencing depth. The sequences contained two scaffolds, a chromosome, and a plasmid. The chromosome of 1,221,268 bp in length contained 1,029 predicted CDSs, 3 rRNAs, 38 tRNAs, 3 ncRNAs, and 9 pseudogenes. Meanwhile, the plasmid of 7,728 bp in length contained 8 predicted CDSs. Information on the genome of the Siamese crocodile *Chlamydia* is shown and compared with that of its closely related species, *C*. *poikilothermis* and *C*. *caviae*, in Table 2.

**Table 2:** Genome comparison of *C*. *crocodili* with its closely related species, *C*. *poikilothermis* and *C*. *caviae*.

	*C*. *poikilothermis*	*C*. *caviae*	*C*. *crocodili*
Accession number	NZ_LS992154.1	NC_003361.3	CP060791
Host	Snake	Guinea pig	Siamese crocodile
Isolate	S15-834K	GPIC	No. 12
Chromosome length (bp)	1,155,104	1,173,390	1,221,268
GC content (%)	37.50	39.22	37.46
Genes	1,040	1,040	1,073
Number of CDSs	989	982	1,029
rRNAs	3	3	3
tRNAs	38	38	38
ncRNAs	3	3	3
Pseudogenes	7	14	9
Plasmid length (bp)	7,559	7,966	7,728
Number of CDSs on plasmid	8	7	8

**Fig 3:**
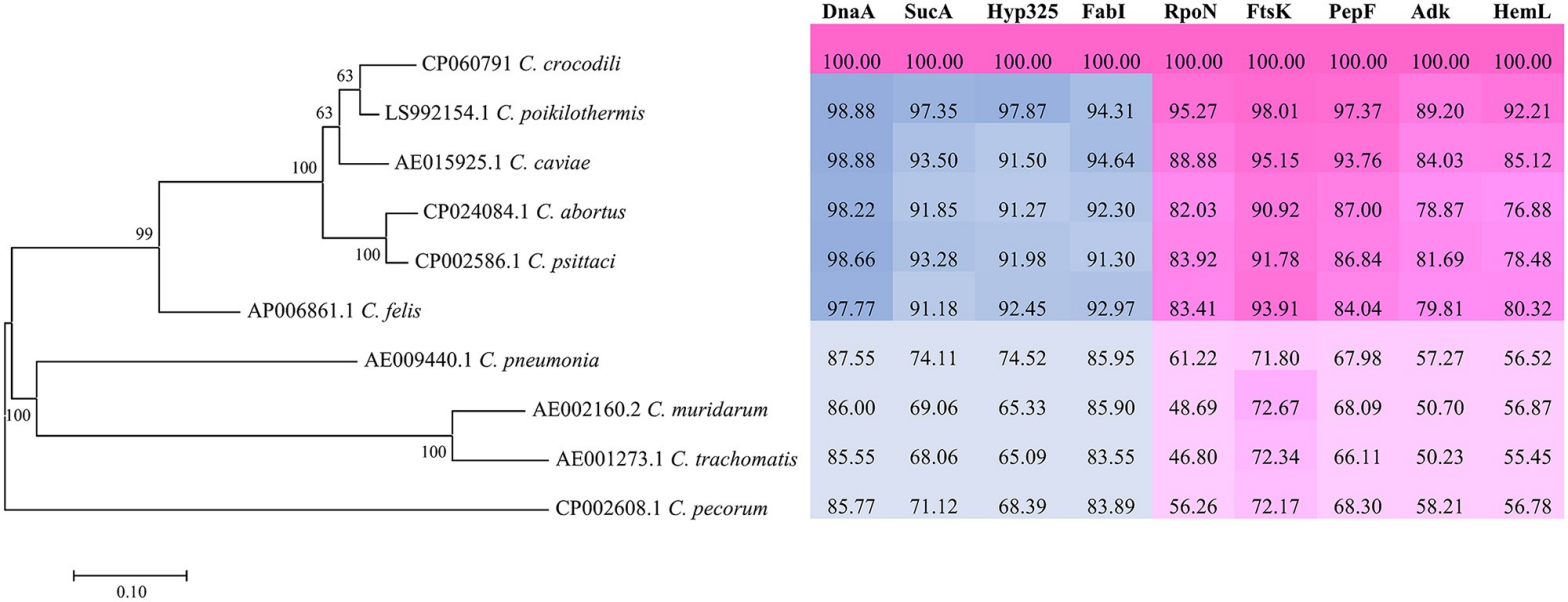
Phylogeny and pairwise amino acid sequence identity of nine taxonomic markers of other *Chlamydia* comparing with *C*. *crocodili*. DnaA, SucA, Hyp325, and Fabl with cut-off values of ≥70%, ≥64%, ≥57%, and ≥78%, respectively, are used to delineate species of the same genus. RpoN, FtsK, PepF, Adk, and HemL with cut-off values of ≤96%, ≤98%, ≤96%, ≤95%, and ≤95%, respectively, are used for new species delineation.

**Fig 4:**
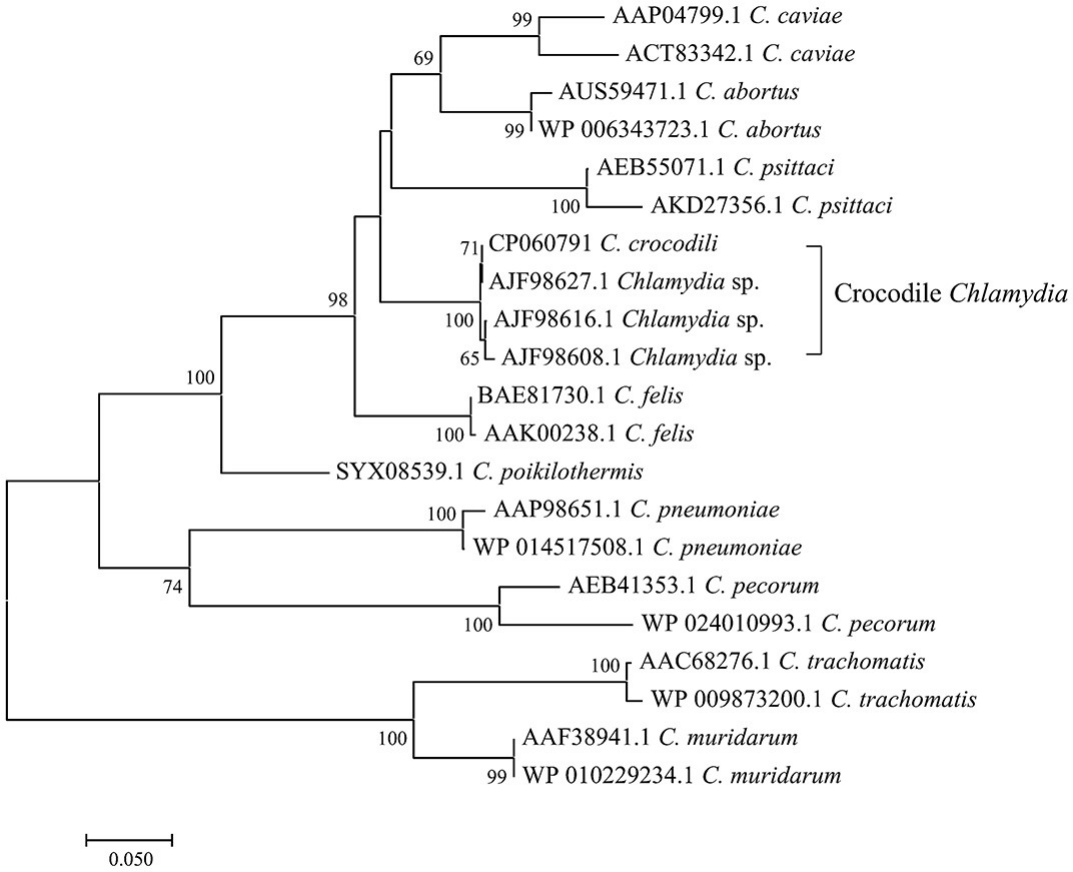
The *ompA* phylogeny.

**Fig 5:**
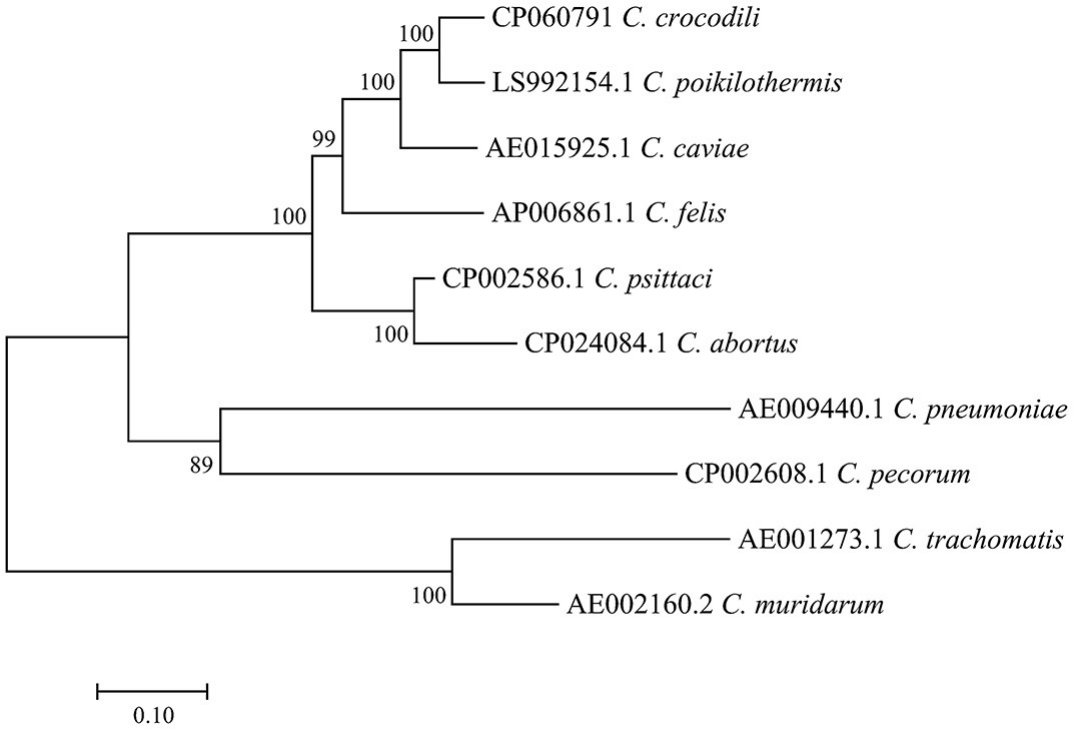
Phylogenetic tree of seven concatenated housekeeping genes.

The full gene analysis of 16S and 23S rRNA of this bacterium demonstrated nucleotide identity to other members of the family *Chlamydiaceae* at rates of 99.74% and 99.76%, respectively (S1 and S2 Tables). It was thus assigned to the family *Chlamydiaceae* (16S and 23S rRNA ≥ 90%) and the genus *Chlamydia* (16S and 23S rRNA ≥ 95%). However, because this strain shares more than 97% 16S and 23S rRNA gene sequence similarity to other *Chlamydia*, species delineation was characterized by other methods with higher resolution. Classification based on the percentage of sequence identity of nine proteins (RpoN, FtsK, PepF, Adk, HemL, DnaA, SucA, Hyp325, and Fabl) [18] was performed. As shown in Fig 3, the percent identity of DnaA, SucA, Hyp325, and Fabl was 98.88%, 97.35%, 97.87%, and 94.34%, confirming that the Siamese crocodile *Chlamydia* is indeed a member of the *Chlamydia* genus. For species delineation, the percentage identity of RpoN, FtsK, PepF, Adk, and HemL was determined to be 95.27%, 98.01%, 97.37%, 89.20%, and 92.21%, respectively. Two out of these five taxonomic markers (FtsK and PepF) did not discriminate it from its closely related species, *C*. *poikilothermis*. Nevertheless, the phylogenetic tree constructed from concatenated sequences of nine protein marker genes showed the distinctness of Siamese crocodile *Chlamydia* from other *C*. *poikilothermis* (Fig 3). The phylogeny constructed from the *ompA* gene and from seven concatenated housekeeping genes also clearly demonstrated the separation between Siamese crocodile *Chlamydia* and *C*. *poikilothermis* (Figs 4 and 5). Similarly, whole-genome-based taxonomic analysis performed at TYGS demonstrated a potential novel species of this strain (S1 Fig). In addition, ANI and dDDH were also used for microbial species delineation by means of genome-to-genome sequence comparison. The values from comparisons between genomes, namely, Siamese crocodile *Chlamydia* versus *C*. *poikilothermis* and Siamese crocodile *Chlamydia* versus *C*. *caviae*, showed 91.74% and 85.98% for ANI, and 46.7% and 31.0% for dDDH, respectively. These values were lower than the cut-off values, namely, 95% for ANI and 70% for dDDH, delineating Siamese crocodile *Chlamydia* from those species. From the overall results, we propose the Siamese crocodile *Chlamydia* as a novel species, which we named *Chlamydia crocodili*.

## Discussion

In Thailand, *Chlamydia* was first detected in Siamese crocodiles in 2012. At that time, the *Chlamydia* taxon was suspected to be a new strain based on 16S rRNA, 23S rRNA, and *ompA* gene analysis [8]. Besides numerical criteria of nucleotide or amino acid sequence identity, the classification of a novel species requires relevant phenotypically distinct features that validate the discrimination [24]. The limitation of cultivation at that time motivated us to emphasize Siamese crocodiles’ *Chlamydia* by *in vitro* isolation. Now, we have successfully cultivated this strain in McCoy cells, allowing more complete characterization. The Siamese crocodile *Chlamydia* was grown at 30°C in McCoy cells, corresponding to the preferred body temperature of freshwater crocodiles, which is in the range of approximately 29°C–33°C [25]. Like all other members of *Chlamydiaceae*, under electron microscopy, this strain was composed of EBs, IBs, and RBs formed in the cytoplasm of infected cells.

Previously, 16S and 23S rRNA gene sequences with cut-offs of 97%, 95%, and 90% nucleotide identity have generally been used to delineate species, genus, and family levels, respectively, of members of the order *Chlamydiales* [26]. From these criteria, Siamese crocodile *Chlamydia* can be assigned to the family *Chlamydiaceae* and the genus *Chlamydia* (99.74% and 99.76% for 16S and 23S rRNA sequence identity, respectively). However, by using 16S and 23S rRNA analysis, Siamese crocodile *Chlamydia* does not meet the recognized classification for species level. Other reports showed that the recognized classification also does not match other closely related strains such as *C*. *psittaci*, *C*. *abortus*, *C*. *felis*, and *C*. *caviae* [27]. Nowadays, whole-genome sequencing is more widely accessible, so 16S and 23S rRNA classification of *Chlamydiaceae* has been replaced by other methods such as determining the percentage identity of nine taxonomic markers, ANI, dDDH, and phylogeny based on the whole genome or marker genes [18, 19, 24, 28–30]. Using a classification scheme based on the percentage of sequence identity of nine conserved taxonomically informative gene products (RpoN, FtsK, PepF, Adk, HemL, DnaA, SucA, Hyp325, and Fabl), all values obtained from Siamese crocodile *Chlamydia* passed the cut-off for species assignment, with the exceptions of FtsK and PepF. The percent identity of FtsK showed a borderline value of 98.01% (cut-off of ≤98%), whereas PepF supported the classification into the same species as *C*. *poikilothermis* with 97.37% identity (cut-off of 96%). However, these conflicting results were overcome by ANI, dDDH, and phylograms based on the whole-genome sequence or marker protein genes. The phylograms of the nine taxonomic markers, seven housekeeping genes, and the *ompA* gene demonstrated separation of the branch of Siamese crocodile *Chlamydia* from *C*. *poikilothermis*. Additionally, the ANI of above 95% and dDDH of more than 70% are recommended to delineate bacterial species [23, 24, 28, 29]. The ANI and dDDH analyses comparing the Siamese crocodile *Chlamydia* genome to those of its most closely related species, *C*. *poikilothermis* and *C*. *caviae*, indicated that the Siamese crocodile *Chlamydia* isolate can be distinguished from those species with ANI values of 91.74% and 85.98% and dDDH values of 46.7% and 31.0%, respectively. As a consequence, we propose classifying the Siamese crocodile *Chlamydia* as a new species to be designated as *Chlamydia crocodili* sp. nov. (No.12).

### Description of *Chlamydia crocodili* sp. nov. (No.12)

*Chlamydia crocodili* (crocodili. L. masc. gen., of the crocodile, because crocodile is the only host currently known). This species was isolated from Siamese crocodiles and may be present in other crocodiles. The bacterium can be recovered from cloacal swabs, pharyngeal swabs, conjunctival swabs, and tissue of internal organs such as liver, spleen, lung, and brain. In terms of the clinical signs of infected crocodiles, they could be asymptomatic, or show kyphoscoliosis in juveniles, conjunctivitis, pharyngitis, ascites, depression, anorexia, and death. Like all other species of *Chlamydiaceae*, *C*. *crocodili* strain can only be grown in cell culture but with an incubation temperature of 30°C. The mode of transmission and zoonotic potential are unknown. The morphology as revealed by electron microscopy showed infected cell forms included in the cytoplasm, having EBs, RBs, and IBs.

## Supporting information

S1 Fig Tree inferred with FastME 2.1.6.1 [1] from GBDP distances calculated from whole-genome sequences. The branch lengths are scaled in terms of the GBDP distance formula d5. The numbers above branches are GBDP pseudo-bootstrap support values > 60% from 100 replications, with average branch support of 68.7%. The tree was rooted at the midpoint [2].
(TIF)

S1 Table 16S rRNA pairwise identity. (XLSX)

S2 Table 23S rRNA pairwise identity. (XLSX)

S1 File References cited in the supporting information. (DOCX)
