# Supplementary figures and images for: A new species of Chlamydia isolated from Siamese crocodiles (Crocodylus siamensis)
Source: PLoS One. 2021 May 27;16(5):e0252081. doi: 10.1371/journal.pone.0252081 (PMC8158970; doi:10.1371/journal.pone.0252081)

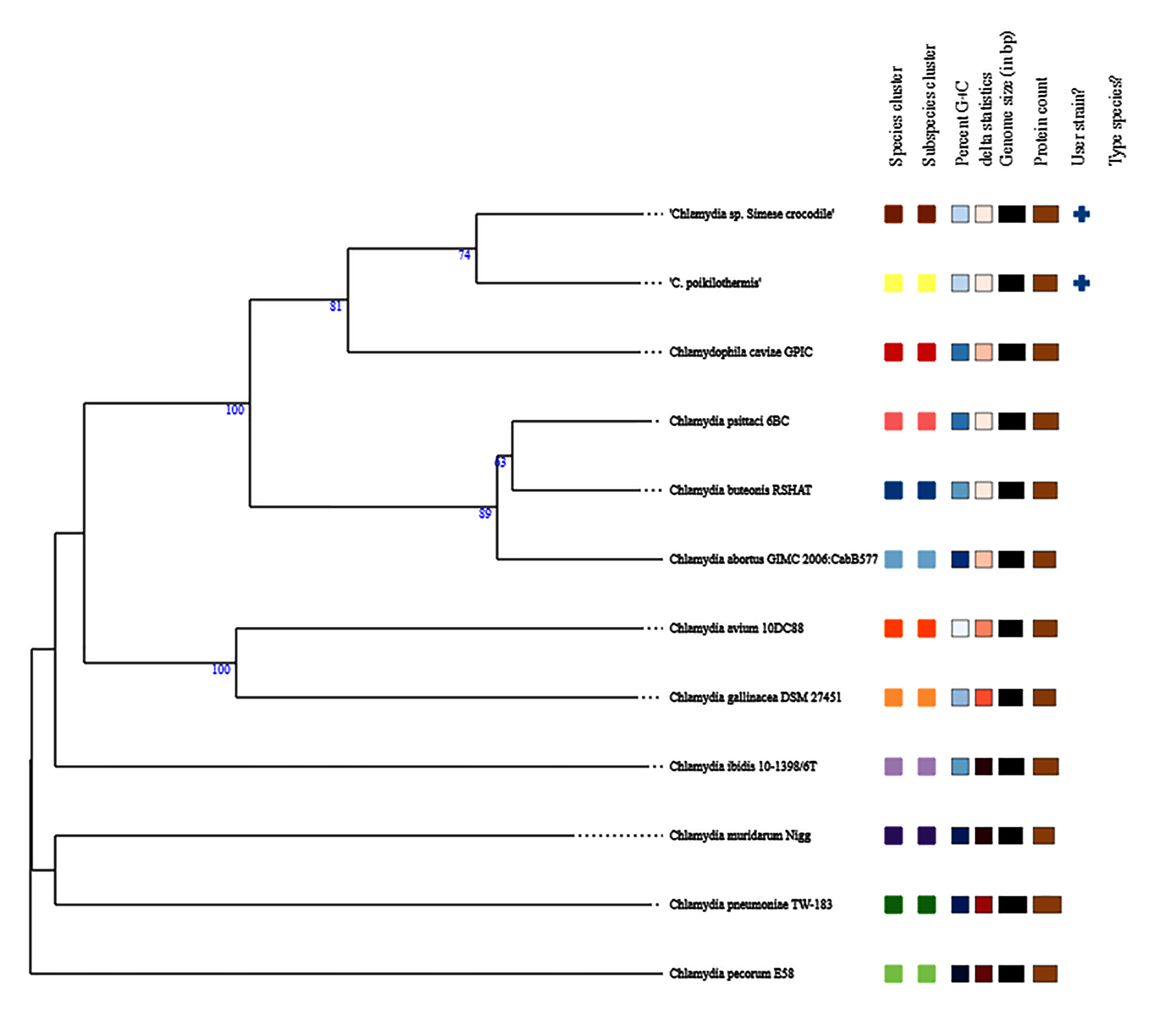

Supplement: S1 Fig — The branch lengths are scaled in terms of the GBDP distance formula d5. The numbers above branches are GBDP pseudo-bootstrap support values > 60% from 100 replications, with average branch support of 68.7%. The tree was rooted at the midpoint [2]. (TIF) [file pone.0252081.s001.tif]
