# Supplementary material for: A new species of Chlamydia isolated from Siamese crocodiles (Crocodylus siamensis)
Source: PLoS One. 2021 May 27;16(5):e0252081. doi: 10.1371/journal.pone.0252081 (PMC8158970; doi:10.1371/journal.pone.0252081)
Supplement: S1 File — (DOCX) [file pone.0252081.s004.docx]

1. Lefort V, Desper R, Gascuel O. FastME 2.0: A Comprehensive, Accurate, and Fast Distance-Based Phylogeny Inference Program. Mol Biol Evol. 2015;32(10):2798-800.

2. Farris J. Estimating Phylogenetic Trees from Distance Matrices. The American Naturalist. 1972;106:645 - 68.
